# An evaluation of direct PCR assays for the detection and quantification of *Porphyromonas gingivalis*

**DOI:** 10.1017/S0950268820001053

**Published:** 2020-05-18

**Authors:** B. L. Gu, Y. J. Qi, J. Y. Kong, Z. T. Li, J. P. Wang, X. Yuan, S. G. Gao

**Affiliations:** 1 Henan Key Laboratory of Cancer Epigenetics; Cancer Institute, The First Affiliated Hospital, and College of Clinical Medicine of Henan University of Science and Technology, Luoyang 471003, China; 2 Medical College of Henan University of Science and Technology, Luoyang 471003, China; 3 Health Management Center, The First Affiliated Hospital, and College of Clinical Medicine of Henan University of Science and Technology, Luoyang 471003, China

**Keywords:** Direct PCR, epidemiological investigation, *Porphyromonas gingivalis*

## Abstract

*Porphyromonas gingivalis* has been linked to the development and progression of oesophageal squamous cell carcinoma (ESCC), and is considered to be a high-risk factor for ESCC. Currently, the commonly used methods for *P. gingivalis* detection are culture or DNA extraction-based, which are either time and labour intensive especially for high-throughput applications. We aimed to establish and evaluate a rapid and sensitive direct quantitative polymerase chain reaction (qPCR) protocol for the detection of *P. gingivalis* without DNA extraction which is suitable for large-scale epidemiological studies. Paired gingival swab samples from 192 subjects undergoing general medical examinations were analysed using two direct and one extraction-based qPCR assays for *P. gingivalis*. Tris-EDTA buffer-based direct qPCR (TE-direct qPCR), lysis-based direct qPCR (lysis-direct qPCR) and DNA extraction-based qPCR (kit-qPCR) were used, respectively, in 192, 132 and 60 of these samples for quantification of *P. gingivalis*. The sensitivity and specificity of TE-direct qPCR was 95.24% and 100% compared with lysis-direct qPCR, which was 100% and 97.30% when compared with kit-qPCR; TE-direct qPCR had an almost perfect agreement with lysis-direct qPCR (*κ* = 0.954) and kit-qPCR (*κ* = 0.965). Moreover, the assay time used for TE-direct qPCR was 1.5 h. In conclusion, the TE-direct qPCR assay is a simple and efficient method for the quantification of oral *P. gingivalis* and showed high sensitivity and specificity compared with routine qPCR.

*Porphyromonas gingivalis*, a common pathogen in human periodontal disease, is considered to be a predictor of disease progression and activity [1]. Recently, several papers have reported that *P. gingivalis* is associated with multiple types of human cancers [2, 3], including oral, oesophageal and pancreatic cancer, as well as some extra oral infection-related diseases [4]. In China, oesophageal squamous cell carcinoma (ESCC) represents one of the major histological subtypes which accounts for approximately half of new cases diagnosed annually worldwide. Previously, we found that the substantial load of *P. gingivalis* in ESCC correlated with differentiation, lymph node metastasis and poor prognosis [2] and the abundance of the organism was significantly higher in ESCC compared with low or undetectable levels in the gastric cardia and distal stomach cancers, respectively [5]. Taken together, these findings suggest that *P. gingivalis* could be a high-risk factor for ESCC, in particular in high endemic areas in China. However, the prevalence of *P. gingivalis* in such populations remains to be characterised, hence the need for a simple, rapid and accurate method for its detection. Currently, molecular and culture techniques remain the major tools for the detection of *P. gingivalis* in periodontal samples. Although highly specific, the culture method requires approximately 5–7 days to positivity, but has low specificity [6], and is poorly suited for molecular epidemiological studies. PCR-based techniques, especially quantitative (qPCR) have been widely used to quantify *P. gingivalis* DNA in saliva, dental plaque and other samples [7]. Most of these assays require DNA extraction or release processes which are not only time-consuming, but also result in loss of template DNA [8].

Direct PCR amplification assays have been widely used for several years for a number of applications and offer multiple advantages, being rapid, economical, efficient and minimises laboratory variation and sample contamination. However, the preservative agents used in oral swabs pose some challenges for PCR detection as they may contain known PCR inhibitors, such as high concentrations of salt and EDTA, as well as detergents [9].

We set out to develop and evaluate the applicability of a Tris-EDTA (TE) buffer based direct qPCR to detect *P. gingivalis* DNA in oral swab samples for large-scale epidemiological studies, and compared this assay with lysis-based direct qPCR and DNA extraction-based qPCR.

Paired oral swabs were taken from 192 subjects undergoing medical examinations at the First Affiliated Hospital of Henan University of Science and Technology in September 2019. Subject exclusion criteria were as follows: brushed teeth in the morning; periodontal or oral treatment in the last year; antibiotics or anti-inflammatory drug therapy in the last 3 months; any related systemic disease (e.g. diabetes.); pregnant; and subjects with less than 20 teeth. The study design was approved by the Ethics Committee of the First Affiliated Hospital of Henan University of Science and Technology, and all participants gave informed consent. Demographic data of subjects included age, sex, smoking status (defined as never, former and current smoking) and alcohol consumption.

The samples were collected in the morning by swabbing the inside of the cheek for 30 s using an iCleanhcy^®^ flocking swab (Shenzhen HuaChenYang Technology Co., Ltd, Shenzhen, China) according to the manufacturer's instruction. The head of the swab was cut-off and placed into the tube with 1 ml of preservative solution in the original packaging; these were stored at 4 °C and tested within a week of sampling.

The collecting tube was vortexed for 30 s. After a brief spin (3500–5000 × g for 1 min), the supernatant was transferred to a 1.5 ml microcentrifuge tube and spun at 12 000 × g for 10 min; the supernatant was discarded.

One set of the 192 samples was treated as follows: 50 μl of Tris-EDTA (10 mm Tris, 1 mm EDTA, pH 8.0) was added, vortexed thoroughly to resuspend the pellet, which was retained at room temperature until use. A second set of 132 from the 192 subjects were subjected to the following treatment: 50 μl of lysis buffer (#18LS11001, Yaneng BioSciences, Shenzhen, China) was added to each swab vortexed thoroughly, microfuged and the pellet was resuspended as above. After heating at 100 °C for 10 min, the extract was centrifuged at 12 000 × g for 10 min, and the supernatant was stored at room temperature until use. For the remaining 60 samples, DNA was extracted using the MicroElute Genomic DNA Kit (#D3096-02, Omega Bio-tek, Inc. Norcross, Georgia., USA), and eluted in 30 μl of preheated Elution Buffer.

The sensitivity of the direct qPCR assay was determined on a simulated positive sample of a 1 ml suspension of *P. gingivalis* ATCC33277 containing 1 × 10^9^ copies/ml; this was added to the prepared pellet of a gingival swab taken from a *P. gingivalis*-negative participant. After centrifuging at 12 000 × g for 10 min, the pellet was resuspended in 100 μl of TE buffer which was then serially diluted in 10-fold steps to give concentrations from 10^7^ to 10 copies/ml. The cycle threshold (Ct) value was used to estimate the cell number per sample.

The primer and probe sequences used in this study were as described previously and synthesised by Genewiz Company, Suzhou, China. To determine an appropriate DNA amplification system for the TE-direct qPCR, 11 *P. gingivalis*-positive swab samples were incorporated into the background of two DNA polymerase systems, namely AceQ qPCR Probe Master Mix (#Q112-03, Vazyme, Nanjing, China) and 2 × Goldstar Master Mix (#CW0939 M, Cowin Bio., Beijing, China). Two master mixes (AceQ and Goldstar) each containing 0.5 μl of primer set (10 μmol), 0.2 pmol of probe and 10 μl of respective master mix were prepared. After vortexing, these were separately aliquoted into PCR tubes and 2 μl of *P. gingivalis*-positive sample was added to each amplification system. Ct values and the relative fluorescence units (RFU) were used to compare the performance of the standard template in each amplification system.

For the quantification of *P. gingivalis* in the sample, each 20 μl of reaction mixture for direct qPCR consisted of 2 μl template/50 ng DNA, 0.5 μl of primer set (10 μmol), 0.2 pmol of probe and 10 μl AceQ qPCR Probe Master Mix. The reaction conditions were 10 min at 95 °C, 40 cycles of 10 s at 95 °C and 60 s at 60 °C. *P. gingivalis* ATCC 33277 at 1 × 10^3^ copies/ml and DEPC water (#R1600, Solarbio, Beijing, China) were used as positive and negative controls, respectively. Amplification was conducted in the CFX96^TM^ Real-Time PCR System (BIO-RAD), and results were analysed by CFX Maestro^TM^ software.

SPSS Statistics 19.0 software (IBM Corporation, Armonk, NY, USA) was used for all data analysis. Data were expressed as percentage (%), mean ± SEM (*x̄* ± *s*). Comparisons were performed using the *χ*^2^ method for categorical data and independent sample *t* tests for quantitative data. *P* < 0.05 was considered statistically significant. Concordance of the results for TE-direct qPCR and lysis-direct qPCR/kit-qPCR from the same individuals were evaluated using % concordance and Cohen's *κ* coefficient. The sensitivity and specificity of the two assays were compared within 95% confidence limits.

Direct qPCR quantification showed the highest reliability in the range of 1 × 10^6^ to 1 × 10^3^copies/ml in the standard concentration curve with a slope of −3.321 (*R*^2^ = 0.997). The amplification efficiency was 100% and Ct values were within 22–32 cycles (Fig. 1A and B). Thus, the limit of detection for *P. gingivalis* was 2 copies/reaction or 1000 copies/ml. A Ct value >38 was defined as negative.

**Fig. 1.**
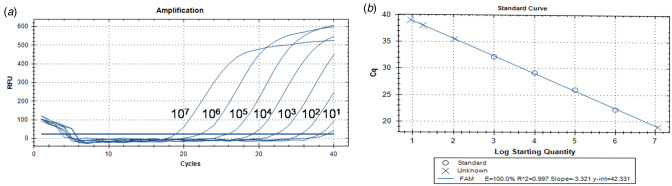
The sensitivity of direct qPCR amplification system for *Porphyromonas gingivalis*. (A) Amplification curve of standard samples. (B) Standard curve.

The 11 *P. gingivalis*-positive samples were amplified in two independent experiments (*n* = 4, *n* = 7), as shown in Figure 2A and B. Note that the Ct value is earlier and the RFU is higher in AceQ qPCR Probe Master Mix compared with 2 × Goldstar Master Mix, even though the target, probe and amplification machine are the same in both cases (Fig. 2A–C). The mean Ct values were 26.10 (19.58–30.51) for 2 × Goldstar Master Mix *vs.* 25.20 (18.57–30.04) for AceQ qPCR Probe Master Mix (*P* < 0.01; Fig. 2D). As a consequence, we chose the latter for further assays.

**Fig. 2.**
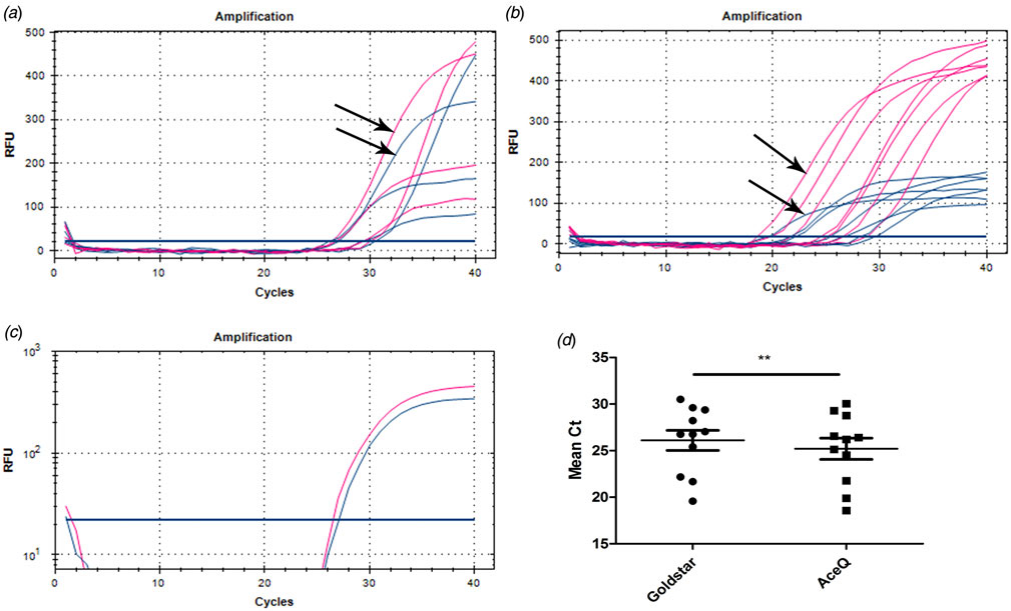
Amplification results of 11 swab samples in different DNA polymerase mixes, AceQ qPCR Probe Master Mix (red lines) and 2 × Goldstar Master Mix (blue lines). The black arrow indicates differences of Ct and RFU values in (A) four samples and (B) seven samples. (C) Amplification log plot in one of the 11 tested samples. (D) Comparison for mean Ct of 11 swab samples between the two master mixes. **P < 0.01.

To compare the PCR systems, one of the paired swabs for each of the 192 participants was subjected to TE-direct qPCR, and the second swab to lysis-direct qPCR (132 samples) or kit-qPCR (60 samples). Table 1a summarises the results of the comparison of TE-direct *vs.* lysis-direct methods and Table 1b shows that of TE-direct *vs.* kit-qPCR methods. The mean log10 copies/ml values were similar, and there were no significant differences in Ct values for both comparisons. Between TE-direct and lysis-direct assays, the positive and negative concordance rates were 97.56% and 97.87%, respectively, with high agreement (97.73%). Similar concordance rates were evident between the TE-direct and kit-qPCR assays, the former showing high sensitivity and specificity compared with either the lysis-direct methods or with the kit-qPCR.

**Table 1.** TE-direct qPCR results of *Porphyromonas gingivalis* compared with (a) lysis-direct qPCR and (b) kit-qPCR

A		
Measurement	Lysis-direct qPCR	TE-direct qPCR
Positive rate (%)	63/132 (47.73%)^a^	60/132 (45.45%)^a^
Mean log10 copies/ml *P. gingivalis* (range)^b^	4.32 (3.01–5.68)^c^	4.46 (3.27–5.97)^c^
Ct mean (range)^b^	28.04 (22.21–33.61)^d^	27.45 (21.08–33.28)^d^
Test measurement (95% CI) of TE-direct qPCR to lysis-direct qPCR		
Sensitivity		95.24%
Specificity		100%
Agreement		97.73%
*κ*		0.954

B		
Measurement	Kit-Qpcr	TE-direct qPCR
Positive rate (%)	23/60 (38.33%)^e^	24/60 (40.00%)^e^
Mean log10 copies/ml *P. gingivalis* (range)^b^	4.74 (3.35–5.72)^f^	4.51 (3.47–6.84)^f^
Ct mean (range)^b^	26.33 (22.33–32.00)^g^	27.26 (17.78–31.53)^g^
Test measurement (95% CI) of TE-direct qPCR to kit-qPCR		
Sensitivity		100%
Specificity		97.30%
Agreement		98.33%
*κ*		0.965

a *χ*^2^ = 0.137, *P* = 0.711.

b Of PCR-positive samples. Paired *t*-test.

c *P* = 0.200.

d *P* = 0.186.

e *χ*^2^ = 0.035, *P* = 0.852.

f *P* = 0.206.

g *P* = 0.207.

The corresponding assay time for the three methods was 1.5 h (TE-direct), 2 h (lysis-direct) and 3 h (kit-qPCR). Furthermore, the costs for TE-direct qPCR before genomic amplification was less than US$0.1, compared with approximately US$0.5 for lysis-direct and US$2.0 for kit-qPCR.

Participants enrolled in this study included 88 males and 104 females with an average age of 43.4 ± 16.9 years. All, but four, of the subjects yielded consistent results by the three assays. Half of all males and those aged ⩾40 years (47.13%) were positive for *P. gingivalis* compared with 39.4% of females aged <40 years (41.6%); these differences were not statistically significant, and similar positivity rates were evident for smokers and non-smokers.

To the authors' knowledge, this is the first study comparing direct qPCR assays for the quantification of *P. gingivalis* in gingival swab samples. Such assays allow the direct amplification of DNA without the need for extraction or releasing techniques. It is reported that the quantity of DNA extracted from saliva by oral swab is limited, and some assay target is lost in the extraction process [8]. Hence, the use of a direct qPCR offers the possibility of enhancing the bacterial target template in a specific, cost-effective and rapid assay (1.5 h) which can be applied for large-scale surveillance of oral pathogens.

For direct qPCR, it is necessary to resuspend cells in a buffer, such as TE [10] to remove preservatives in the swab likely to inhibit the reaction. As the DNA polymerase system is a key factor for the optimisation of direct qPCR, we compared the performance of two such systems in pilot samples and found that the AceQ qPCR Probe Master Mix gave superior results over the 2 × Goldstar solution in terms of amplification capability and relative signal intensity; this was therefore chosen for subsequent testing using the *P. gingivalis* ATCC 33277 specific primer and probe for the construction of the TE-direct qPCR assay.

Some inconsistent results were evident in our study in that three samples proved positive by the lysis-direct qPCR but negative by TE-direct qPCR. Retests of these samples ranged from weak positive to negative results and this was attributed to possibly low levels of target DNA or the presence of an inhibitor. Only one case was positive by TE-direct qPCR in duplicate tests but negative by kit-qPCR.

This study has a number of limitations. A significant weakness is that we did not collect data on the oral health of the subjects. Our testing protocol requires further optimisation to clarify technical parameters such as the range of volume of TE buffer for solubilisation and standardisation of cell deposits across samples. In addition, we did not investigate whether the three discordant results were due to PCR inhibitors. Despite these shortcomings, we found the TE-direct qPCR to be a rapid assay with good sensitivity and specificity. In conclusion, the TE-direct qPCR assay which does not require template extraction or release showed good potential for large-scale quantitative screening of *P. gingivalis* in oral swabs when compared with two other qPCR assays.
